# The Prevalence of Zinc Deficiency in Patients With Thalassemia in South East of Iran, Sistan and Baluchistan Province

**DOI:** 10.5812/ircmj.6243

**Published:** 2014-08-05

**Authors:** Mohammad Ali Mashhadi, Zahra Sepehri, Zahra Heidari, Eghbal Shirzaee, Zohre Kiani

**Affiliations:** 1 Department of Hematology and Oncology, Ali Ebne Abitaleb Hospital, Zahedan University of Medical Sciences, Zahedan, IR Iran; 2 Department of Internal Medicine, Amir Al-Momenin Hospital, Zabol University of Medical Sciences, Zabol, IR Iran; 3 Department of Internal Medicine, Ali Ebne Abitaleb Hospital, Zahedan University of Medical Sciences, Zahedan, IR Iran; 4 Kerman University of Medical Sciences, Kerman, IR Iran

**Keywords:** Thalassemia Major, Zinc, Iran

## Abstract

**Background:**

There are different and controversial reports about zinc deficiency in patients with major thalassemia.

**Objectives:**

The aim of this study was to evaluate zinc status in patients with major thalassemia in Sistan and Baluchistan province, southeastern Iran.

**Patients and Methods:**

The study was performed in Ali Asghar Hospital, a specialized governmental hospital located in Zahedan, Iran. In this cross-sectional study, 369 patients with a history of major thalassemia for more than 5 years entered the study using convenience sampling method. Thirty-six subjects were excluded from the study based on our exclusion criteria. Zinc level was measured in all patients after 12 hours fasting using atomic absorption spectrometry method in 2012.

**Results:**

Of 369 cases, 333 patients were eligible and evaluated. The mean age was 15.63 ± 7.4 years. One hundred ninety two cases were male and others were female (141 cases). About 27% (90) of the cases were 5-10 years-old, 24% (80) were 10-15 years-old and 49% were older than 15 years old. Iron chelator in 65.46% was Desferrioxamine, in 28.2% was Deferasirox and in 19.5% was combination of Desferrioxamine and Deferiprone. All cases had zinc deficiency, and 98.5% had severe zinc deficiency. Others (1.5%) had mild deficiency.

**Conclusions:**

Our study on 333 patients with major thalassemia documented severe zinc deficiency in all cases. We had no cases with normal or increased zinc levels. It was different with other reports in the world.

## 1. Background

Thalassemia is the most common hereditary anemia (1, 2). Iran is among countries with highest prevalence of major thalassemia (3). This severe hereditary anemia needs lifelong blood transfusion. According to this modality of treatment, multiple problems such as metabolic, endocrine and growth abnormalities would occur (4, 5). Chronic blood transfusion in major thalassemia or other hemolytic disorders such as Sickle cell anemia may decrease chronic hemolysis and change the micronutrient status (6-8). Zinc is a trace element involved in synthesis of many other properties such as cholesterol, fat and more than 300 enzymes and immune and antioxidant system (9, 10). Zinc has essential role in red blood cell survival and zinc deficiency leads to membrane fragility (11). Multiple nutrient products contain zinc. High levels of zinc exist in oysters, red meat, poultry, beans, nuts, certain seafood, whole grains, fortified breakfast cereals, and dairy products. Vegetables, fruits, tea, coffee, rice and bread have low levels of zinc (12).

## 2. Objectives

This study aimed to evaluate zinc status in a large group of patients with major thalassemia.

## 3. Patients and Methods

This cross-sectional study was performed on patients with major thalassemia in Ali Asghar Hospital in Sistan and Baluchistan province, southeastern of Iran, between February and September of 2012. Ali Asghar is a specialized governmental hospital with 60 beds in Zahedan. The study was approved in ethics committee of Zahedan University of Medical Sciences (zaums.REC.1390.32). The sample size was calculated as 234 cases (power = 80%, α = 0.05, confidence interval = 95%, the disease prevalence = 64). Inclusion criteria were age above 5 years, documented major thalassemia (with hemoglobin electrophoresis), transfusion dependency, and normal kidney and liver function tests. Exclusion criteria were known liver disease, kidney disease, gastrointestinal disease, specific diet habit such as vegetable regimen, and consumption of zinc and minerals. After 12 hours fasting, blood samples were collected. All blood samples were centrifuged after 45 minutes spontaneous coagulation and then stored at -20ºC. Zinc level was measured using graphite furnace atomic absorption spectrometry Varian, Australia (spectr AA 240fs, 2009, USA). Weights and heights were measured by Hitec (model HI-72, Canada). One observer collected data. Data entered SPSS and reported using mean and standard deviation. Statistical analysis was performed using t-test, ANOVA and chi-square tests. Central limit theorem was considered for using parametric test because of a large sample size in subgroups and also values of standard deviation compared to the mean values.

## 4. Results

From all patients (369 cases) with major thalassemia older than 5 years, 333 cases were eligible and evaluated for zinc status. Demographic, hematological and biochemical parameters of patients were demonstrated in Table 1. The mean age of male and female cases were 15.52 ± 5.99 and 15.78 ± 9.16 years old, respectively. The mean BMI was 16.55 ± 3.31 kg/m^2^. The mean duration of blood transfusion was 11.48 ± 15.39 months and the mean duration of iron chelator therapy was 64.64 ± 47.03 months. The mean ferritin level was 4053.162 ± 2523.058 μg/mL. All of eligible cases (333 cases) had decreased serum zinc level; 98.5% (328 cases) had severe zinc deficiency (based on standards not in comparison with our general population) and 1.5% (5 cases) had mild zinc deficiency. Mean zinc levels are reported in Table 2 according to patients’ characteristics and type of chelator.

The mean zinc level was 18.36 ± 10.33 µg/dL. The mean value in males was 17.85 ± 10.10 µg/dL and 19.07 ± 10.62 µg/dL in females (P = 0.0001). Mean zinc levels according to patients’ characteristics are showed in Table 3. The mean zinc status according to the type of iron chelator was not significantly different (P < 0.946). The mean duration of packed RBC transfusions was 2.48 ± 1.06 months.

**Table 1.** Demographic, Hematological and Biochemical Parameters of Patients With Thalassemia

Variables	Patients With Thalassemia No. (%)
**Gender**	
Male	192 (57.7)
Female	141 (42.3)
**Age**	
5-10 years-old	90 (27)
10-15 years-old	80 (24)
> 15 years-old	163 (49)
**Iron chelator**	
Desferal	218 (65.6)
Deferasirox	65 (19.5)
Desferal and L1	94 (28.2)

**Table 2.** Zinc Status According to Patients' Characteristics

	Sever Zinc Deficiency No. (%)	Mild Zinc Deficiency No. (%)	P Value
**Sex**			0.642
Male	189 (98.4)	3 (1.6)	
Female	139 (8.6)	2 (1.4)	
**Age**			0.123
5-10 years-old	90 (100)	0 (0)	
10-15 years-old	77 (96.3)	3 (3.7)	
> 15 years-old	161 (98.8)	2 (1.2)	
**BMI**			0.686
< 20 kg/m^2^	285 (98.3)	5 (1.7)	
20-25 kg/m^2^	37 (100)	0 (0)	
25-30 kg/m^2^	6 (100)	0 (0)	
**Iron chelator**			0.985
Desferal	150 (98)	3 (2)	
Deferasirox	95 (98.9)	1 (1.1)	
Desferal and L1	64 (98.5)	1 (1.1)	

**Table 3.** Mean Zinc Status According to Patients’ Characteristics

Variables	Mean	P Value
**Gender**		0.0001
Female	19.7 ± 10.62	
Male	17.85 ± 10.10	
**Age**		0.147
5-10	18.17 ± 9.47	
10-15	20.28 ± 11.77	
> 15	17.53 ± 9.96	
< 20	18.32 ± 10.42	
**Body Mass Index**		0.288
20-25	19.55 ± 10.26	
25-30	14.23 ± 3.32	
**Iron chelator**		0.946
Deferral	18.22 ± 10.68	
Deferasirox	17.95 ± 16.05	
Desferal and L1	17.69 ± 9.24	

## 5. Discussion

This large cross-sectional study on 333 cases with major thalassemia showed a high prevalence (100%) of zinc deficiency; most patients had severe deficiency (98.5%). Mild zinc deficiency was detected in 1.5% of cases. Many authors reported zinc deficiency in these patients by searching the literature. In those studies, the number of patients with thalassemia was not comparable to our study and age distribution was also different. In fact, the most important finding in this large study was severe zinc deficiency in near all cases, which was different with other studies.

In the study of Claster et al. (6), 43 cases with sickle cell anemia and 24 cases with major thalassemia were evaluated. The mean age was 14.5 years; iron chelator in most patients was deferasirox. Zinc deficiency was found in 24.3% and 8.3% of patients with sickle cell anemia and major thalassemia. Some possible reasons for these differences may be wider age distribution and different types of iron chelator. Another reason may be due to the method of measurement. Measuring plasma zinc level helps to diagnose major deficiencies, despite the fact it is quite insensitive to marginal deficiency because a change in plasma zinc does not occur until zinc intake is extremely low.

Therefore, a patient with normal results may still be deficient. In fact, one possible reason for difference is power of the test in detecting severe but not moderate or mild deficiencies (13).

In other study in Tehran (14), 220 cases with thalassemia were studied. The mean age was 15.2 ± 3.1 years similar to our study. The mean BMI was 18.3 ± 3 kg/m^2^. This finding is also similar to our study. The mean zinc level was 54.6 ± 4 µg/dL and the prevalence of zinc deficiency was 79.6%. This finding was different with our results. This difference may be due to diet of our patients, since the major diet of our patients was wheat products. Wheat cannot supply sufficient zinc. On the other hand, drinking tea after meals and frequently may affect the results. It is recommended to consider these probabilities in further studies (13).

One study on Jordanian patients with major thalassemia reported a high level of zinc in 42 cases which was significant (P < 0.05) (15). This study showed no differences between both sexes. Our study was in a larger group of patients (333 cases) and age distribution was 5-33 years, and we reported main changes in zinc status in different types of iron chelator. In this study, zinc status was high. In this study, in contrast to our study, gender was not a significant parameter. One possible reason for this different finding of our study is power of the study; probably reporting no difference between both sexes is due to low power of the mentioned study. Another study on 61 cases with major thalassemia according to the type of iron chelator reported that in Desferal group 50% of cases had zinc deficiency and this was 32.8% in Desferal and deferiprone group (16). Small sample size and low power of the study, and weakness of the test in detecting mild and moderate deficiencies are possible reasons of the difference. In the study of Mahyar conducted on 40 cases, the mean zinc level was 67.35 ± 20.38 µg/dL (17). Twenty-six percent of the cases had moderate zinc deficiency (65%) and no statistical significance was found between zinc deficiency and ferritin level, BMI, sex, age, blood transfusion and iron chelator therapy. Compared to our study, small number of patients, different inclusion criteria especially in age distribution, different populations and maybe diet habits are possible causes for this discrepancy. Another study (18) reports the incidence of zinc deficiency in 10% of patients with major thalassemia different to near 100% prevalence of our study. One of the main reasons for different results is method of determining zinc level between these studies. Moafi et al. measured hair zinc level, which is influenced by few factors such as some hair products (18). It raises again the question about actual zinc status in patients with thalassemia, so we discuss it in our study limitations. In other study in Tehran thalassemia center (19), 131 cases were evaluated and results were similar to our study. The mean age was 15.2 ± 3 years and the mean BMI was 17.7 ± 3.5 kg/m^2^. Zinc deficiency was found in 85.5% of cases. This finding was significantly different in males and females. Serum zinc was higher in females (P < 0.0001). In this study, results were similar to ours. Dehshal et al. reported zinc deficiency in 38% of patients in thalassemia and a relation between zinc level and abnormal serum glucose, which was different form our results (20). The difference may be due to different criteria for sampling, since Dehshal et al. selected patients with transfusion-related iron overload and adult patients. This may alter zinc status and make the difference. Another study in Iraq (21) was performed on 105 cases with major thalassemia whose age range was 2.5-18 years. The study showed significant zinc deficiency; the mean zinc status was 38.55 ± 20.25 µg/dL. This finding was different to our results since the mean zinc level in our study was lower than 18.36 ± 10.33 µg/dL. In another study on 72 cases of Indian patients with thalassemia, authors showed significant zinc deficiency with a mean zinc level of 46.97 ± 8.40 µg/dL, which was different to our study (22). Different sampling method, different culture and maybe diet habits were some possible causes for the difference.

Our study had some limitations. We reported severe zinc deficiency in about all of our major thalassemia patients, but this may reflect zinc deficiency in all of the Iranian general population. Therefore, we need a population-based study to determine zinc status in a general population in Iran to interpret the results appropriately. In fact, we need to know the range of zinc level in general population using the same method.

On the other hand, 85% of total body zinc is intracellular and less than 0.1% of body zinc appears in the plasma. The level of plasma zinc is dependent on the continuous shift between intracellular sources and intestinal absorption. Because of these reasons, plasma zinc is a poor indicator for total body zinc. Metabolic stress, which is frequent in patients with thalassemia may increase the intracellular shift of zinc and decrease plasma level in case of unchanged total body zinc. Zinc redistribution confounds interpretation of low plasma zinc concentrations (23). In these circumstances, biomarkers of metabolic zinc redistribution are needed to determine whether redistribution or poor nutrition is the cause of low plasma zinc. Therefore, we suggest measurement of metallothionein or cellular zinc transporters to answer the questions about zinc status in such patients.

Zinc status in most studies had low level and mild to moderate zinc deficiency was the most common finding in patients with major thalassemia. The strong point of this study was its large sample size and wide age distribution. Our study showed that about all patients with thalassemia had severe zinc deficiency. It is important to know when to initiate complementary regimen for patients with zinc deficiency. Finally, it is recommended to perform other case-control studies to evaluate the effect of dietary regimen on zinc status.
